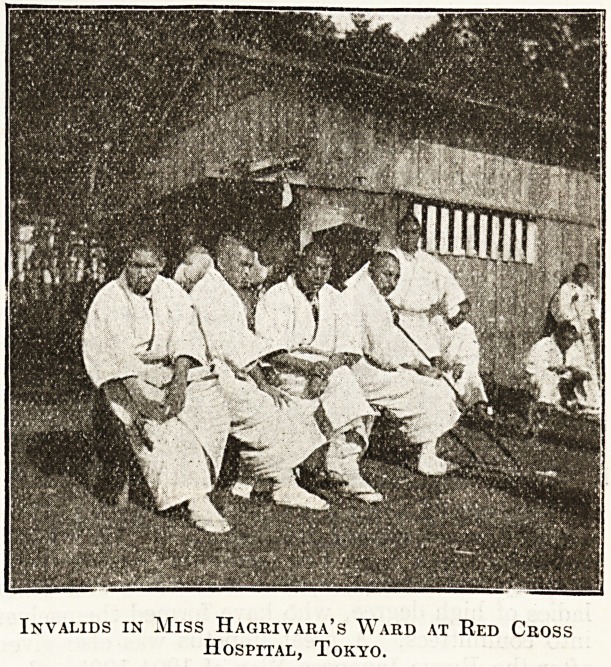# The Red Cross Society of Japan During Peace and War. By a Life Member

**Published:** 1912-10-26

**Authors:** 


### THE RED CROSS SOCIETY OF JAPAN.

### During Peace and War. By a Life Member.

The Dowager Empress Haruka, the patroness-in-chief of the Red Cross Society, takes a keen and personal interest in all that concerns the practical arrangements connected with its work, and the Society owes its phenomenal success chiefly to Her

The Operating Room of the Central Red Cross Hospital at Tokyo.

Majesty and to the Imperial Princesses and other ladies of high degree, who have formed themselves into committees. A great impetus was also given after the Russo-Japanese War of 1904-1905, when Japan faced the problem of what was to be done with her superfluous women, for in thousands of homes the breadwinner did not return, and throughout the Empire women were forced to work for their living as they had never done before.

The Central Hospital of the Red Cross Society at Shibuya, Tokyo, is a large, handsome building on the model of the University Hospital in Heidelberg, Germany, and it is admirably adapted for the purpose it serves, with nineteen wings built out independently of each other, overlooking charming gardens on which tired eyes rest gratefully. In time of peace it is used for the purpose of training physicians, surgeons, and women nurses; and all sorts and conditions are treated there, the poor being free from charges and the rich paying due expenses. In time of war it is utilised as the reserve hospital of the Army, and during the last campaign the Empress visited it frequently. The nurses, who are admitted between the ages of seventeen and thirty, remain there for three years, beginning as students and passing out as graduates eligible to take private cases, but liable to be called on for service. They are, therefore, enrolled as reserve nurses of the Society under vow, which means that they take a solemn oath in writing to keep themselves for fifteen years ready to respond at any time to the first call of the Society, for service in time of war, political disturbances, or for instruction manœuvres and similar purposes.

The Red Cross Society secures physicians by paying the expense of educating a number of medical students, on condition that they become reserve physicians on their graduation. After some years' practice in the Central Hospital in Tokyo, those who are really efficient are sent to Europe to perfect their studies—again at the expense of the Society—so that there are always one or two of these doctors staying in Europe, and bringing back to their beloved country the latest discoveries of medical science. Physicians bind themselves by vow in the same manner as nurses for a fixed period of five years.

The limits of age, after which one is considered incapable of service, are fifty-five for physicians, pharmacists, and nurses, and forty-five for attendants and stretcher-bearers. All the members of the relief staff are paid, not only for service, but also for adhering to the vow; and they receive travelling expenses, in addition to their excellent salary, when they are summoned in time of war, or even for manœuvres. If they contract illness or receive wounds in consequence of the service, pensions are paid to them, which go to their families in case of death.

One important reason for the efficiency of the medical service of the Japanese Army during the [illegible]

Ward of Tokyo Reserve Hospital.

Russo-Japanese War is to be found in the fact that in time of peace the Red Cross Society of Japan takes a real part in the manœuvres of the Army, large and small. On such occasions the Relief Staff is sent out exactly as in the case of actual war—they establish hospitals on the supposed line of communication, and they receive and cure not only mock patients, but also real ones suffering from illness or wounds received during the manœuvres. Thus they obtain, in time of peace, a thorough knowledge of the part they are to play in time of war, in organising the whole medical service of the Army, an experience which is quite apart from hospital training; and, when the manœuvres are over, criticisms are made upon their conduct by the Chief Medical Officer of the Army, and valuable suggestions offered.

When, therefore, on the outbreak of the Russo-Japanese War the call "to arms" resounded through the country the Red Cross Society, with its splendid organisation, was ready for any emergency. After years of quiet and steady progress its methods were almost perfect, for Japan had made a study of these in the best foreign medical schools, and had skilfully adapted them to her own requirements. There was no confusion, no lack of help, no breakdown in the transmission of stores and medicines to the front, each department being supervised by experienced officials.

Without doubt one of the chief reasons for the success of the Japanese in that war was their attention to minute detail, a quality which they carried with them from everyday life, and which was invaluable in hospital work, where every item was carefully considered, and nothing left to chance. I remember, for instance, that when I helped to make warm belts and caps for the soldiers written directions were given as to the size and amount of wool to be used in each.

It was the rule of the Japanese surgeons at the front to do little or no operating except in cases of extreme emergency, or where hæmorrhage threatened immediate death. The Hospital Corps men at the front had been trained in the most thorough and practical manner in Tokyo, and all cases were treated by the application of first-aid dressings, and then sent to the rear as quickly as possible, then by hospital boat or transport to the base hospitals in Japan. The result was that many of the wounded needed nothing but medical treatment after their arrival, hundreds of bullet wounds having been healed by first attention after the non-operative treatment at the front; and it was thus proved that the modern bullet is absolutely aseptic, where the lesion is uncomplicated. Thousands of lives were saved in this manner through the Hospital Corps of the Red Cross Society.

Invalids in Miss Hagrivara's Ward at Red Cross Hospital, Tokyo.

There is a general belief that the Japanese do not feel pain as much as Europeans, and possibly this idea is due to their undoubtedly strong nerves. They are much less afflicted with headaches, and our modern ailments, neurasthenia and nervous breakdown, are unknown; even when they feel ill their natural self-control makes them give as little trouble as possible to others, as those who have nursed them can testify. Among country people in distant villages the Society is popularised by illustrated lectures with magic-lantern views, which show the rescue work of the Society not only in war, but also in times of public calamity, such as earthquakes, eruptions, tidal waves, conflagrations, inundations, hurricanes; and also shipwrecks, railway disasters, and accidents in the crowding of people on public occasions.

Accident Ward of Matsuyama Hospital.

## Figures and Tables

**Figure f1:**
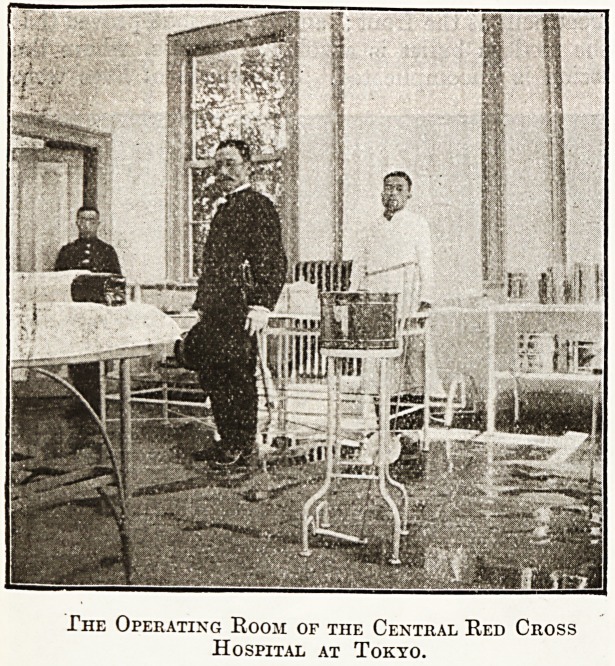


**Figure f2:**
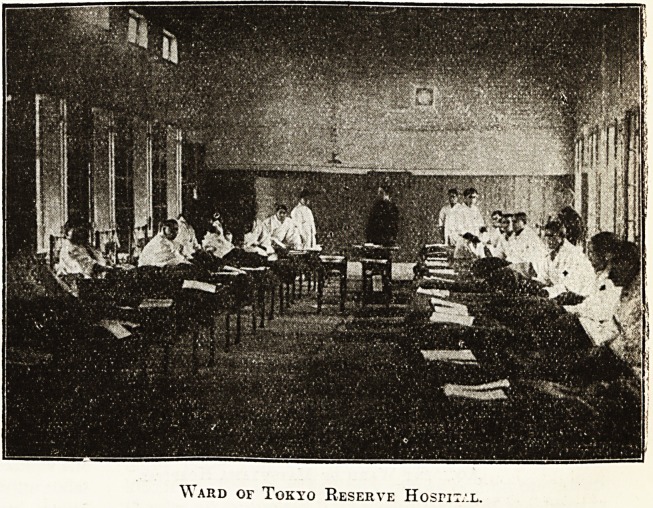


**Figure f3:**
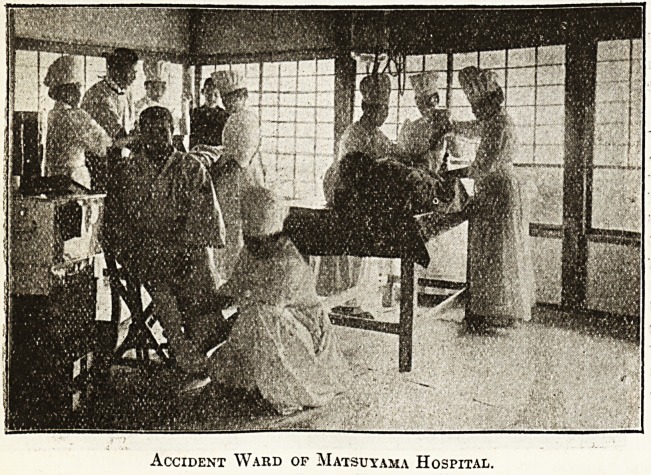


**Figure f4:**